# Assessment of demographic bias in retinal age prediction machine learning models

**DOI:** 10.3389/frai.2025.1653153

**Published:** 2025-10-10

**Authors:** Christopher Nielsen, Emma A. M. Stanley, Matthias Wilms, Nils D. Forkert

**Affiliations:** ^1^Department of Radiology, University of Calgary, Calgary, AB, Canada; ^2^Biomedical Engineering Graduate Program, University of Calgary, Calgary, AB, Canada; ^3^Hotchkiss Brain Institute, University of Calgary, Calgary, AB, Canada; ^4^Alberta Children’s Hospital Research Institute, University of Calgary, Calgary, AB, Canada; ^5^Department of Radiology, University of Michigan, Ann Arbor, United States; ^6^Department of Clinical Neurosciences, University of Calgary, Calgary, AB, Canada

**Keywords:** retinal age prediction, machine learning, bias, multimodal imaging, retinal imaging

## Abstract

The retinal age gap, defined as the difference between the predicted retinal age and chronological age, is an emerging biomarker for many eye conditions and even non-ocular diseases. Machine learning (ML) models are commonly used for retinal age prediction. However, biases in ML models may lead to unfair predictions for some demographic groups, potentially exacerbating health disparities. This retrospective cross-sectional study evaluated demographic biases related to sex and ethnicity in retinal age prediction models using retinal imaging data (color fundus photography [CFP], optical coherence tomography [OCT], and combined CFP + OCT) from 9,668 healthy individuals (mean age 56.8 years; 52% female) in the UK Biobank. The RETFound foundation model was fine-tuned to predict retinal age, and bias was assessed by comparing mean absolute error (MAE) and retinal age gaps across demographic groups. The combined CFP + OCT model achieved the lowest MAE (3.01 years), outperforming CFP-only (3.40 years) and OCT-only (4.37 years) models. Significant sex differences were observed only in the CFP model (*p* < 0.001), while significant ethnicity differences appeared only in the OCT model (*p* < 0.001). No significant sex/ethnicity differences were observed in the combined model. These results demonstrate that retinal age prediction models can exhibit biases, and that these biases, along with model accuracy, are influenced by the choice of imaging modality (CFP, OCT, or combined). Identifying and addressing sources of bias is essential for safe and reliable clinical implementation. Our results emphasize the importance of comprehensive bias assessments and prospective validation, ensuring that advances in machine learning and artificial intelligence benefit all patient populations.

## Introduction

Retinal imaging has become a valuable, non-invasive data source for studying aging and ocular and systemic health (Li and Lin, 2024). Due to its shared embryological origins with the central nervous system, the retina can reflect vascular and neurodegenerative changes that are otherwise difficult to assess. High-resolution retinal imaging enables the direct visualization of microvasculature and neural tissue, offering insights into ocular, cardiovascular, and neurological health. Recent advances in machine learning (ML) have enabled the estimation of biological retinal age from retinal images, leading to the concept of the retinal age gap, which denotes the difference between predicted retinal age and true chronological age (Nielsen et al., 2025a; Nielsen et al., 2025b). Recent studies suggest that increased retinal age gaps may indicate elevated risk for stroke, Parkinson’s disease, and mortality, supporting a shift toward biological age markers as more accurate indicators of health than chronological age alone (Grimbly et al., 2024).

Despite these promising results, concerns are growing regarding the fairness and generalizability of ML tools across diverse populations (Norori et al., 2021). For example, if an ML model underestimates retinal age in certain demographic groups, clinicians may misjudge disease risk, leading to potentially missed diagnoses and exacerbated health disparities. Previous literature has shown how biases from imbalanced demographics, spurious correlations, or mismatches between training and deployment populations can reduce model reliability (Jacoba et al., 2023). As ML becomes more common in ophthalmology, understanding how such biases can influence predictive performance is critical. For example, Burlina et al. (2021) showed that imbalanced training sets can reduce diagnostic consistency in diabetic retinopathy detection, while Lin et al. (2023) found that ML models may misclassify glaucoma in underrepresented groups. However, it remains unclear whether and to what extent retinal age prediction models may be affected by biases leading to unfair biological age predictions for different demographic groups.

In this work, we exemplarily investigate bias differences in the context of ML models trained on two commonly used retinal imaging modalities: color fundus photography (CFP) and optical coherence tomography (OCT). More specifically, we evaluate how RETFound, a widely used retinal image analysis model performs for retinal age prediction across these modalities and examine whether differences in model prediction errors exist within two demographic factors, namely sex and ethnicity.

## Materials and methods

### Dataset

We utilized data from the UK Biobank, a large population-based repository of participants who underwent detailed health assessments, including retinal imaging (Bycroft et al., 2018). Our final cohort comprised 9,668 participants, selected through a multi-step filtering process designed to ensure data quality and create a healthy cohort for model training and evaluation. First, we performed rigorous quality control on both CFP and OCT images to remove scans with motion artifacts or any other significant acquisition issues. CFP image quality was assessed using a deep learning-based method proposed by Fu et al. (2019), while OCT images were evaluated using the image quality score provided by the Topcon Advanced Boundary Segmentation (TABS) algorithm, as described by Chen et al. (2024). A primary inclusion criterion was the availability of a matched pair of high-quality CFP and OCT images for each participant. Following this, we excluded participants with any self-reported medical conditions, based on the criteria developed by Zhu et al. (2023). Finally, only images from the right eye of each unique participant were included in the final dataset. This approach was chosen to maximize the number of individuals in our cohort, as requiring high-quality images from both eyes would have significantly reduced the sample size. Additionally, using only one eye per participant avoids the need for statistical correction for within-subject inter-eye correlation. In the next step, self-reported ethnicities with less than 200 participants were excluded to ensure robust statistical analyses. The dataset was split into training (50%), validation (10%), and test (40%) sets, stratified by age, sex, and self-reported ethnicity to ensure balanced representation. Demographics for each split are shown in Table 1.

**Table 1:** Demographic information for participants included in the training, validation, and test sets.

Characteristic	Training	Validation	Test
Age (mean ± SD)	52.9 ± 8.0	52.9 ± 8.1	52.9 ± 8.0
Sex			
Female	54.0% (2,612)	54.0% (522)	54.1% (2,092)
Male	46.0% (2,222)	46.0% (444)	45.9% (1,776)
Ethnicity			
White	94.0% (4,544)	94.0% (908)	94.0% (3,635)
Asian	3.0% (144)	3.0% (29)	3.0% (115)
Black	3.0% (146)	3.0% (29)	3.1% (118)

The dataset was stratified to ensure similar distributions of age, sex, and ethnicity across all three sets.

### Model architecture and training

We employed the publicly available RETFound foundation model, which was previously successful in many retinal image analysis tasks (Zhou et al., 2023). Model weights were fine-tuned for retinal age prediction based on the RETFound authors’ guidelines across three configurations: (1) CFP only, (2) OCT only, and (3) combined CFP + OCT. The combined approach used late-fusion, concatenating single-modality representations before the final layer. Fine-tuning was used to minimize mean squared error loss between predicted and chronological age, using the Adam optimizer with early stopping based on validation loss. Training was conducted in PyTorch 1.13.1 on an NVIDIA RTX 3090 GPU.

### Statistical analysis

Retinal age prediction performance was evaluated using mean absolute error (MAE) between predicted biological age and chronological age. To assess demographic bias, we adapted the approach by Piçarra and Glocker (2023), previously applied to assess sex and ethnicity bias in brain age prediction. Kruskal-Wallis tests were used to compare retinal age gaps across sex and ethnicity groups. To correct for multiple comparisons (three model types, two subgroup categories), we applied a Bonferroni-adjusted significance threshold of $\alpha =\frac{0.05}{6}$ .

## Results

The combined CFP + OCT model yielded the lowest overall MAE (3.01 years), outperforming the CFP-only (3.40) and OCT-only (4.37) models (Table 2). Sex-based performance varied: the combined model showed minimal difference (females: 2.98; males: 3.04), CFP slightly favored males (3.34 vs. 3.45), and OCT favored females (4.11 vs. 4.67). The combined model also achieved the lowest MAE across ethnic groups: White (3.01), Asian (2.75), and Black (3.16). Kruskal–Wallis tests revealed significant sex bias in the CFP model (*p* < 0.001), but not in the OCT (*p* = 0.798) or combined models (*p* = 0.019; not significant after correction). Ethnicity bias was significant in OCT (*p* < 0.001), but not in the CFP (*p* = 0.032) or combined models (*p* = 0.131).

**Table 2:** Model performance, measured by mean absolute error (MAE) in years, and bias analysis results.

Characteristic	CFP	OCT	Combined
Overall MAE	3.40	4.37	3.01
Sex			
Female	3.45	4.11	2.98
Male	3.34	4.67	3.04
*p*-value	<0.001*	0.798	0.019
Ethnicity			
White	3.41	4.37	3.01
Asian	3.24	4.10	2.75
Black	3.34	4.45	3.16
*p*-value	0.032	<0.001*	0.131

MAE values are reported for the overall test set and stratified by sex and ethnicity for each of the three models (CFP-only, OCT-only, and combined CFP + OCT). *p*-values are from Kruskal–Wallis tests comparing retinal age gaps between demographic subgroups for each model. The asterisk (*) indicates statistical significance after applying a Bonferroni correction for multiple comparisons.

## Discussion

The results of this study highlight that finetuning ML models for retinal age prediction can result in significant performance differences between sex and ethnicity groups. This aligns with prior work in ophthalmic imaging artificial intelligence, where models for classifying conditions like age-related macular degeneration, diabetic retinopathy, and glaucoma have shown performance disparities between demographic groups (Luo et al., 2024). These findings highlight the importance of evaluating and understanding biases before clinical deployment of ML models. An unrecognized bias could have downstream effects on disease detection and patient care. Thus, thorough bias analyses and prospective validation across diverse populations are of paramount importance (Krause, 2024).

Our findings further demonstrate that combining multiple imaging modalities may improve predictive performance while helping to reduce bias. The CFP + OCT model achieved the lowest MAE, indicating superior accuracy, and showed no significant differences between sex or ethnicity groups. A possible explanation for these results is that CFP and OCT introduce different sources of bias, where CFP was significantly associated with sex-related bias, while OCT showed significant bias related to ethnicity. We hypothesize that these modality-specific biases may reflect true biological differences in retinal aging across demographic groups, which are captured uniquely by each imaging modality. For example, a recent study by Böttger et al. suggested that sex-specific retinal vascular traits can be detected in CFP images (Böttger et al., 2025). Furthermore, Varma et al. (1994) found that males have 2–3% larger optic discs than females, measurable via CFP. Similarly, Wagner-Schuman et al. (2011) and Poon et al. (2018) reported ethnicity-related differences in central retinal thickness detectable using OCT. Therefore, by leveraging data from both modalities, the combined CFP + OCT model may gain a more comprehensive bias-free understanding of retinal aging, overcoming the biases of the single modality approaches. However, further research is necessary to explore multimodal strategies as a means of enhancing fairness in retinal ML models and to better understand the origins of these biases. For instance, it may be argued that the predictive power of our fine-tuned models may rely heavily on the foundational feature representations learned by the RETFound model during its extensive pretraining. It is plausible that the pretraining dataset enabled RETFound to learn feature representations that are more robust or discriminative for predicting age in certain groups, contributing to the observed performance differences. Future work could incorporate visual interpretability methods, such as saliency maps, to identify the specific retinal features driving these predictions and better understand the anatomical basis of model bias (Stanley et al., 2022).

While this study offers valuable insights into bias in retinal age prediction models, certain limitations warrant further investigation. Notably, the UK Biobank predominantly consists of Caucasian participants, with a limited representation of other ethnic groups. Additionally, our definition of a healthy cohort relies on the absence of self-reported disease. Although this aligns with previous UK Biobank retinal age studies (Zhu et al., 2023; Zhang et al., 2023; Hu et al., 2022), this approach may unintentionally include participants with undiagnosed or subclinical conditions that could affect the performance of the retinal age prediction models. Furthermore, our sample size was also constrained by the availability of matched, high-quality CFP and OCT images from healthy participants within a single visit. Moreover, this study relied on self-reported ethnicity, which is an interpretable but broad categorization. Future research could benefit from correlating prediction errors with genetic principal components to uncover ancestry-related associations that might be overlooked by discrete categories. Additionally, exploring socioeconomic factors as potential biases in retinal age prediction models would be beneficial for future studies. Finally, while this work offers valuable insights based on a large UK-based population cohort, external validation is essential. Broadening the analysis to encompass more diverse datasets and additional machine learning architectures will further strengthen the generalizability of the results.

## Conclusion

This work demonstrates that imaging modality selection (CFP *vs.* OCT *vs.* combined) affects both performance and bias profiles of retinal age prediction models. As the retinal age gap emerges as a promising biomarker for disease detection, understanding and mitigating bias sources is crucial for safe, reliable implementation. Our findings underscore the need for thorough bias analyses and prospective evaluation to ensure ophthalmic artificial intelligence advancements benefit all patient populations.

## Data Availability

The data analyzed in this study is subject to the following licenses/restrictions: accessing the UK Biobank requires registration. Requests to access these datasets should be directed to https://www.ukbiobank.ac.uk/enable-your-research/apply-for-access.
